# Immunophenotypic characterization of acute leukemias in Bahia, Brazil

**DOI:** 10.31744/einstein_journal/2023AO0117

**Published:** 2022-12-15

**Authors:** Mariane Melo dos Santos, Allan Souza dos Santos, Herbert Henrique de Melo Santos, Lorene da Silva Santos, Roberto José Meyer Nascimento, Alex José Leite Torres

**Affiliations:** 1 Universidade Federal da Bahia Salvador BA Brazil Universidade Federal da Bahia, Salvador, BA, Brazil.

**Keywords:** Leukemia, Immunophenotyping, Acute myeloid leukemia, Precursor B-cell lymphoblastic leukemia-lymphoma, Flow cytometry, Leukemia, myeloid, acute

## Abstract

**Objective:**

To characterize the immunophenotypic profile of acute leukemias in the population of the state of Bahia, Brazil.

**Methods:**

This is a descriptive, retrospective study. From 2014 to 2018, 796 new cases of acute leukemia were evaluated. The data were obtained from analysis of reports and records of tests performed by flow cytometry immunophenotyping. All individuals of all age groups diagnosed as acute lymphoblastic leukemia or acute myeloid leukemia were included in the study. Demographic variables and expression of leukemia antigens were evaluated.

**Results:**

Most cases were diagnosed as acute myeloid leukemia and 42.7% as acute lymphoblastic leukemia. Significant differences were found in expression of markers in acute leukemias when age groups were compared, as well as in demographic characteristics. B-cell acute lymphoblastic leukemia was more prevalent than cases of T-cell origin. Assessing the aberrant markers in acute myeloid leukemias, the non-acute promyelocytic leukemia group presented expression of CD7 and CD56 as the most frequent ones. In B-cell acute lymphoblastic leukemia, the most frequent aberrant markers were CD66c, CD13 and CD33.

**Conclusion:**

Significant differences were found as to several antigens when comparing adults and children, and these findings may contribute to future studies correlating the phenotypic profile to genetic characteristics and therapeutic response, including specific antigen therapies, which may be better targeted.

## INTRODUCTION

Acute leukemia can be classified into two main groups: acute myeloid leukemia (AML) or acute lymphoblastic leukemia (ALL). The latter is the most common malignant disease in childhood, but it is rare in older individuals, accounting for approximately 15% of leukemias in adults. The incidence of leukemia in Brazil is 5.15 per 100,000 inhabitants.^(1)^ Acute leukemia progresses rapidly and is usually fatal, but treatments have improved significantly in recent years, increasing overall survival and in some cases leading to a cure.^(2-4)^

Acute promyelocytic leukemia (APL), or AML with t(15;17) promyelocytic leukemia (*PML)* retinoic acid receptor α *(RARA)* is characterized by the predominant presence of abnormal promyelocytes. This subtype often presents disseminated intravascular coagulation and increased fibrinolysis, which results in high rates of early mortality; therefore, to make a quick and accurate diagnosis is essential for maintenance of patient’s life. If diagnosed quickly, APL has a favorable prognosis with good responses to treatment.^(5-7)^

Leukemias have complex genetic characteristics, hindering the initial analysis of different genes involved in the disease. This makes diagnosis difficult, negatively interfering in the initial stages of therapeutic approaches.^(8,9)^

Multiparametric flow cytometry immunophenotyping is a standard and essential procedure for diagnosis of acute leukemias. This method identifies cell characteristics, including size, internal complexity and cell antigens; thus, it is possible to establish diagnosis, define leukemia lineage and subclassification, and, in some cases, predict prognosis.^(10,11)^

Leukemia-associated phenotypic markers, commonly known as aberrant markers, are useful to discriminate between normal and reactive precursor cells of leukemic cells. This aberrant labeling occurs when myeloid lineage markers are expressed in lymphoid cells, or when markers of lymphoid origin are expressed in myeloid cells. The occurrence of aberrant markers is reported in acute leukemias with varying frequency and its prognostic value is controversial.^(12,13)^

The regulatory mechanisms related to this phenomenon have been studied. Some hypotheses were raised emphasizing the possible lineage indecisiveness or poor genetic programming, but the biological function of this aberrant expression has not been fully understood yet.^(14,15)^ From a clinical and laboratory point of view, it is crucial to establish associations with chromosomal alterations and prognostic factors, employing more specific molecular tests. In addition, aberrant immunophenotypes are known to be relevant tools to detect minimal residual disease.^(14,16,17)^

Demographic characteristics, such as age, sex and race, have been used to evaluate the risk of leukemia. Individuals aged 1 to 10 years have a favorable prognosis, while the age groups <1 year and >10 years have an adverse prognosis. The white race presents a favorable factor in relation to the black race. Female individuals have a more favorable prognosis.^(18)^

The literature currently lacks data on immunophenotypic characterization in Brazil. Characterizing the immunophenotypic profile of leukemias evaluated in a diagnostic center in the state of Bahia will enable collecting data that may contribute to better targeting therapies in the future and, consequently, to improve the clinical status and survival of these patients.

## OBJECTIVE

To characterize the immunophenotypic profile of acute leukemias in the population of the state of Bahia, Brazil.

## METHODS

This is a descriptive, retrospective study with data obtained from January 2014 to December 2018. The variables of sex, age and immunophenotype were obtained from the analysis of reports and records of tests performed using flow cytometry immunophenotyping. The study was approved by the Research Ethics Committee of *Instituto de Ciências da Saúde da Universidade Federal da Bahia*, under CAAE: 91088418.0.0000.5662; # 2.917.200.

### Study population

All individuals of all age groups diagnosed as ALL or AML and seen at the Laboratory of Immunology and Molecular Biology of *Instituto de Ciências da Saúde da Universidade Federal da Bahia*, in the period between January 2014 to December 2018 were included in the study. Recurrence and reassessment cases were excluded from the study. The Laboratory delivers services to patients by means of the Brazilian Public Health System (SUS - *Sistema Único de Saúde*). Individuals from all microregions of Bahia were analyzed, except those from the microregion of Porto Seguro, because no patient resided in that region.

### Analyzed variables

The panel of antibodies used to define diagnosis of leukemia was comprehensive and also specific for each type of leukemia, as follows:

ALL B: CD19 PECY7, CD20 V450, CD10 APC, CD34 PERCPCY 5.5, CD13 PE, CD33 APC, CD38 APCH7, CD66c PE, CD45 V500, CD79a PE and CD81 FITC.

ALL T: CD3m APCH7, CD3cit V450, CD4 V450, CD8 FITC, CD7 APC, CD2 V450, CD5 PERCPCY 5.5, CD1a APC, CD13 PE, CD33 APC, CD34 PERCPCY 5.5, CD45 V500 and CD117 PECY7.

AML: CD2 FITC, CD7 APC, CD13 PE, CD15 FITC, CD19 PECY7, CD33 APC, CD34 PERCPCY 5.5, CD56 PE, CD64 PE, CD117 PECY7, HLA-DR V450 and MPO FITC.

The analyses were performed on peripheral blood and bone marrow samples. Age and sex variables were also evaluated.

### Statistical analysis

Data were compiled in Microsoft Excel 2016 (Windows 10) spreadsheets and transferred to SPSS Statistics version 25.0 to obtain the descriptive epidemiology of frequencies, and the medians and means were calculated. Furthermore, the data were submitted to Pearson’s χ^2^ statistical test to assess categorical variables.

## RESULTS

From 2014 to 2018, a total of 796 new cases of acute leukemia in the state of Bahia were evaluated; in that, 456 (57.3%) were diagnosed as AML and 340 (42.7%) as ALL. A total of 80 cases were identified as APL, accounting for 17.6% of AML cases and 10.05% of all cases. The other AML subtypes were grouped and named non-APL AML, accounting for 47.24% (n=376) of total cases. Frequency of 42.7% (n=340) was observed when evaluating the ALL, and 277 cases (81.5%) were of B-lymphocyte origin and 63 (18.5%) of T-lymphocyte origin.

The pediatric population (<16 years) comprised 236 cases (78.3% of lymphoid leukemias). In adolescent and adult population (n=560) the frequency of ALL was 27.6%. The peak incidence of B-cell ALL occurred in children aged between 2 and 5 years. In T-cell ALL, the peak incidence was observed in patients aged between 12 and 18 years, and, in AML, it was in those aged above 60 years. The median age of AML was 43 years, and most patients were female 53.2% (p=0.236). The characteristics of the study population, including age, sex, white blood cell count, mean number of blasts and type of sample analyzed, are summarized in table 1.

**Table 1:** Characteristics of the study population

Characteristics	ALL B (n=277)	ALL T (n=63)	Non-APL AML (n=376)	APL (n=80)
Age, n (%)				
≤2 years	22 (7.9)	0 (0)	15 (4)	0 (0)
3-16 years	137 (49.5)*	26 (41.3)	26 (6.9)	10 (12.5)
>16 years	118 (42.6)	37 (58.7)	335 (89.1)*	70 (87.5)*
Median	11	18	52	33
Sex, n (%)				
Female	138 (49.8)	14 (22.2)*	200 (53.2)	42 (52.5)
Male	139 (50.2)	49 (77.8)*	176 (46.8)	38 (47.5)
White blood cell count, n (%)				
<10,000	29 (20)	3 (9)	37 (18)	4 (9)
10,000-50,000	55 (38)	14 (42)	72 (35)	23 (49)
50,000-100,000	29 (20)	7 (21)	43 (21)	11 (23)
>100,000	31 (22)	9 (27)	56 (27)	9 (19)
Mean	81,389	83,639	80,218	60,185
Median	39,500	47,600	44,650	44,700
Blasts, (%)				
Mean	71	62	50	85
Median	77	70	46	88
Sample, (%)				
Bone marrow	69	35	44	60
Peripheral blood	31	65	56	40

* p<0.01. χ^2^ statistical test.

ALL: acute lymphoblastic leukemia; AML: acute myeloid leukemia; APL: acute promyelocytic leukemia.

Markers were evaluated and separated into positive, partially positive and negative expression, grouped by type of leukemia. The cutoff point for marker expression to be considered positive or partially positive was 20% and 10%, respectively. Table 2 summarizes the pattern of expression of these antigens in number of cases and frequency.

**Table 2:** Pattern of antigen expression in leukemia groups

	Non-APL AML			n		APL		n	B-cell ALL			n	T-cell ALL					n
			
	Positive	Negative	Partial		Positive	Negative	Partial		Positive	Negative	Partial		Positive	Negative		Partial		
			
	cases (%)	cases (%)	cases (%)		cases (%)	cases (%)	cases (%)		cases (%)	cases (%)	cases (%)		cases (%)	cases (%)		cases (%)		
CD13	287 (77)	22 (6)	63 (17)	372	74 (93)	0 (0)	6 (7)	80	57 (21)	184 (67)	35 (13)	276	10 (16)		40 (63)	13 (21)	63	
CD33	309 (84)	17 (5)	43 (12)	369	78 (99)	0 (0)	1 (1)	79	29 (11)	204 (74)	43 (16)	276	6 (10)		47 (75)	10 (15)	63	
CD34	222 (60)	93 (25)	58 (15)	373	0 (0)	66 (83)	14 (17)	80	145 (53)	63 (23)	66 (24)	274	11 (18)		32 (52)	18 (30)	61	
CD45	369 (98)	2 (1)	3 (1)	374	80 (100)	0 (0)	0 (0)	80	139 (50)	46 (17)	92 (33)	277	62 (98)		0 (0)	1 (2)	63	
MPO	181 (49)	112 (30)	77 (21)	370	80 (100)	0 (0)	0 (0)	80	0 (0)	277 (100)	0 (0)	277	0 (0)		63 (100)	0 (0)	63	
CD19	7 (1.9)	349 (93.1)	19 (5.1)	375	0 (0)	80 (100)	0 (0)	80	272 (98.2)	1 (0.4)	4 (1.4)	277	0 (0)		63 (100)	0 (0)	63	
CD3cyt	0 (0)	374 (100)	0 (0)	374	0 (0)	79 (100)	0 (0)	79	0 (0)	275 (100)	0 (0)	275	56 (88.9)		0 (0)	7 (11.1)	63	
CD3mem	0 (0)	375 (100)	0 (0)	375	0 (0)	80 (100)	0 (0)	80	0 (0)	276 (100)	0 (0)	276	24 (38.1)		18 (28.6)	21 (33.3)	63	
CD15	10 (3)	334 (90)	25 (7)	369	0 (0)	78 (97)	2 (3)	80	NR	NR	NR		NR		NR	NR		
CD56	13 (7)	151 (82)	21 (11)	185	1 (3)	27 (84)	4 (13)	32	NR	NR	NR		NR		NR	NR		
CD64	67 (18)	262 (70)	43 (12)	372	21 (27)	28 (35)	30 (38)	79	NR	NR	NR		NR		NR	NR		
HLA-DR	271 (72)	47 (13)	45 (15)	375	0 (0)	72 (92)	7 (8)	79	NR	NR	NR		NR		NR	NR		
CD2	9 (2.4)	348 (94.1)	13 (3.5)	370	6 (7.6)	52 (65.8)	21 (26.6)	79	NR	NR	NR		42 (66.7)		11 (17.5)	10 (15.9)	63	
CD7	59 (15.8)	234 (62.6)	81 (21.7)	374	1 (1.3)	78 (97.5)	1 (1.3)	80	NR	NR	NR		62 (98.4)		0 (0)	1 (1.6)	63	
CD117	309 (82.8)	29 (7.8)	35 (9.3)	373	43 (54.4)	5 (6.3)	31 (39.2)	79	NR	NR	NR		8 (2.7)		51 (81)	4 (6.3)	63	
CD10	NR	NR	NR		NR	NR	NR		234 (84.5)	23 (8.3)	20 (7.2)	277	12 (19.7)		34 (55.7)	15 (24.6)	61	
CD20	NR	NR	NR		NR	NR	NR		64 (25.8)	93 (37.5)	91 (36.7)	248	NR		NR	NR		
CD4	NR	NR	NR		NR	NR	NR		NR	NR	NR		14 (22.6)		29 (46.8)	19 (30.6)	62	
CD8	NR	NR	NR		NR	NR	NR		NR	NR	NR		30 (48.4)		19 (30.6)	13 (21)	62	
CD5	NR	NR	NR		NR	NR	NR		NR	NR	NR		49 (77.8)		7 (11.1)	7 (11.1)	63	
CD1a	NR	NR	NR		NR	NR	NR		NR	NR	NR		8 (13.1)		36 (57.1)	17 (27.8)	61	
CD38	NR	NR	NR		NR	NR	NR		123 (74.1)	18 (10.8)	25 (15.1)	166	NR		NR	NR		
CD66c	NR	NR	NR		NR	NR	NR		26 (38.8)	25 (37.3)	16 (23.9)	67	NR		NR	NR		
CD81	NR	NR	NR		NR	NR	NR		93 (93)	2 (2)	5 (5)	100	NR		NR	NR		
CD79a	NR	NR	NR		NR	NR	NR		260 (94.9)	4 (1.5)	10 (3.6)	274	NR		NR	NR		

NR: not rated; cyt: cytoplasmic; mem: membrane; AML: acute myeloid leukemia; APL: acute promyelocytic leukemia; ALL: acute lymphoblastic leukemia.

Chronic myeloid leukemia (CML) can progress to acute leukemia and is called CML blast crisis.^(5)^ In the study, 13 cases of acute leukemia secondary to chronic myeloid leukemia were evaluated. The previous diagnosis of CML was reported in the medical record. Of these, 7 (53.8%) evolved to AML and 6 (46.2%) to B-cell ALL (p=0.782), and 9 (69.2%) were male and 4 (30.8%) female (p=0.267). The median age was 56 years.

### Acute myeloid leukemia

Evaluating the aberrant markers in non-APL AML, CD7 was more frequent (37.4%), followed by CD56 (18.4%). CD19 was expressed in 1.9% of cases and CD2 in 5.9%. Regarding APL, CD7 was found in only 2.5% of cases, whereas CD56 was in 15.6% of cases. CD2 showed a higher frequency, with expression in 34.2% of cases; no expression of CD19 was observed (Table 3).

**Table 3:** Rate of positivity of marker expression in non-APL acute myeloid leukemia and acute promyelocytic leukemia

	non-APL AML	APL	non-APL AML		APL		non-APL AML		APL	
			
			Female	Male	Female	Male	≤15 years	>15 years	≤15 years	>15 years

	Cases/total (%)	Cases/total (%)	Cases/total (%)	Cases/total (%)	Cases/total (%)	Cases/total (%)	Cases/total (%)	Cases/total (%)	Cases/total (%)	Cases/total (%)
CD13	350/372 (94.1)	80/80 (100)	186/197 (94.4)	164/175 (93.7)	42/42 (100)	38/38 (100)	30/36 (83.3)	320/336 (95.2)*	9/9 (100)	71/71 (100)
CD33	352/369 (95.4)	79/79 (100)	189/194 (97.4)	163/175 (93.1)	41/41 (100)	38/38 (100)	36/37 (97.3)	316/332 (95.2)	9/9 (100)	70/70 (100)
CD34	280/373 (75.1)	14/80 (17.5)	143/200 (71.5)	137/173 (79.2)	9/42 (21.4)	5/38 (13.2)	29/37 (78.4)	251/336 (74.7)	1/9 (11.1)	13/71 (18.3)
CD2	22/370 (5.9)	27/79 (34.2)	7/198 (3.5)	15/172 (8.7)*	18/42 (42.9)	9/37 (24.3)	4/35 (11.4)	18/335 (5.4)	1/9 (11.1)	26/70 (37.1)
CD7	140/374 (37.4)	2/80 (2.5)	72/198 (36.4)	68/176 (38.6)	2/42 (4.8)	0/38 (0)	16/37 (43.2)	124/337 (36.8)	0/9 (0)	2/71 (2.8)
CD117	344/373 (92.2)	74/79 (93.7)	184/199 (92.5)	160/174 (92)	40/42 (95.2)	34/37 (91.9)	32/37 (86.5)	312/336 (92.9)	6/9 (66.7)	68/70 (97.1)
CD56	34/185 (18.4)	5/32 (15.6)	19/103 (18.4)	15/82 (18.3)	1/17 (5.9)	4/15 (26.7)	4/15 (26.7)	30/170 (17.6)	0/3 (0)	5/29 (17.2)
CD64	110/372 (29.6)	51/79 (64.6)	57/197 (28.9)	53/175 (30.3)	27/41 (65.9)	24/38 (63.2)	14/37 (37.8)	96/335 (28.7)	6/9 (66.7)	45/70 (64.3)
HLA-DR	328/375 (87.5)	7/79 (8.9)	176/199 (88.4)	152/176 (86.4)	5/42 (11.9)	2/37 (5.4)	29/37 (78.4)	299/338 (88.5)	0/9 (0)	7/70 (10)
MPO	258/370 (69.7)	80/80 (100)	144/197 (73.1)	114/173 (65.9)	42/42 (100)	38/38 (100)	19/37 (51.4)	239/333 (71.8)*	9/9 (100)	71/71 (100)

* p<0.05.

AML: acute myeloid leukemia; APL: acute promyelocytic leukemia.

**Figure 1:**
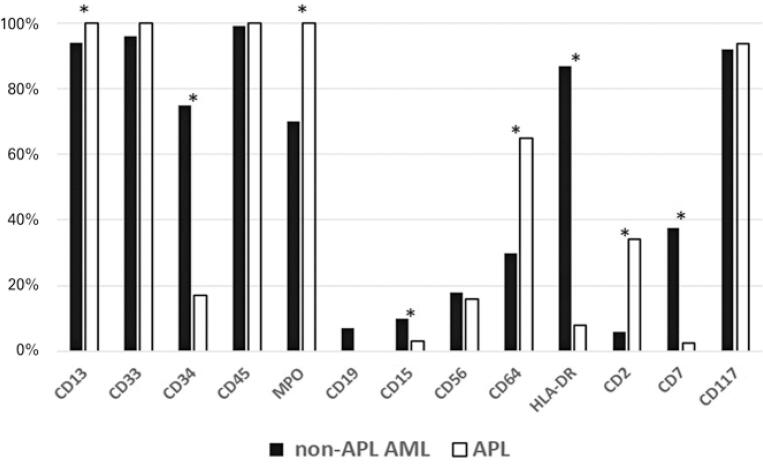
Difference in marker expression of antigens in acute myeloid leukemia

Figure 1 shows the main phenotypic differences between non-APL AML and APL. Based on this analysis, it was possible to determine CD34, HLA-DR, CD13, MPO, CD15, CD64, CD2 and CD7 as the main markers differentiating these two AML groups. The positivity rate was established by adding the cases that had positive and partially positive expression.

* p <0.05.

APL: acute promyelocytic leukemia; AML: acute myeloid leukemia.

**Figure 2:**
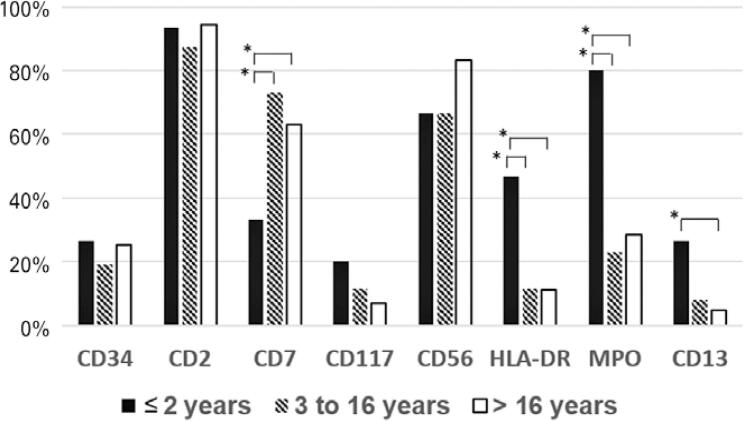
Percentage of cases of non-APL acute myeloid leukemia with negative expression of antigens by age group

Table 3 shows the positivity rate in the non-APL and APL AML groups, separated by sex and age group. In non-APL AML, males showed a significant difference in CD2 expression (p<0.05). Adults had a higher positivity rate in expression of CD13 and MPO (p<0.05). In APL, CD117 was significantly more frequent in adults. When the group of infants (≤2 years) was evaluated separately, in addition to the differences in CD13 and MPO, a significant difference was found in the expression of CD7 and HLA-DR (Figure 2).

* p<0.05.

### Acute lymphoblastic leukemia

A total of 340 ALL cases were evaluated; in that, 277 (81.5%) were B-cell ALL and 63 (18.5%) T-cell ALL. As to sex, 50.2% and 77.8% of cases were male in B-cell ALL and T-cell ALL, respectively (p<0.01).

**Figure 3:**
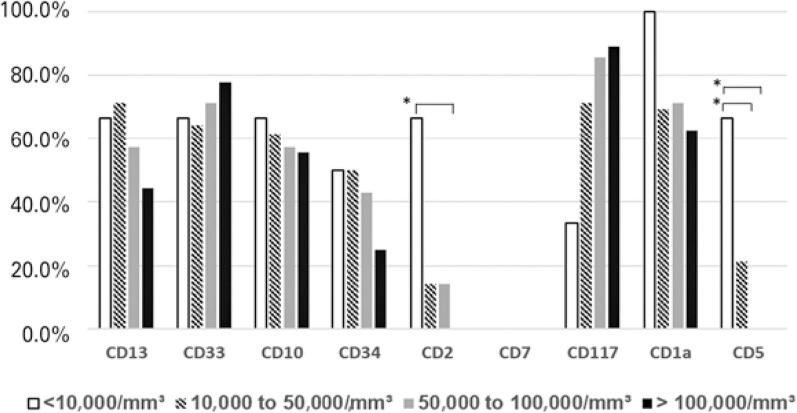
Percentage of T-cell ALL cases with negative expression of antigens by white blood cell count range upon diagnosis

The most frequent aberrant marker in B-cell ALL was CD66c, with a positivity rate of 62.7%. CD13 and CD33 accounted for 33% and 26%, respectively. Table 4 shows the positivity rate in B-cell ALL and T-cell ALL groups, separated by sex and age groups. In B-cell ALL, the frequency of cases with positive expression of CD66c and CD34 was significantly different between adults and children. In T-cell ALL, this difference was seen in the expression of CD2, CD117 and CD5 (Table 4). When evaluating the antigenic expression in the white blood cell count groups, there is a significant difference in the expression of CD2 and CD5 (Figure 3).

**Table 4:** Rate of positivity of expression of markers in B-cell ALL and T-cell ALL

	B-cell ALL	T-cell ALL	B-cell ALL		T-cell ALL		B-cell ALL		T-cell ALL	
			
			Female	Male	Female	Male	≤15 years	>15 years	≤15 years	>15 years

	Cases/total (%)	Cases/total (%)	Cases/total (%)	Cases/total (%)	Cases/total (%)	Cases/total (%)	Cases/total (%)	Cases/total (%)	Cases/total (%)	Cases/total (%)
CD13	92/276 (33)	23/63 (36.5)	43/137 (31.4)	49/139 (35.3)	6/14 (42.9)	17/49 (34.7)	47/152 (30.9)	45/124 (36.3)	8/22 (36.4)	15/41 (36.6)
CD33	72/276 (26)	16/63 (25.4)	34/137 (24.8)	38/139 (27.3)	2/14 (14.3)	14/49 (28.6)	34/152 (22.4)	38/124 (30.6)	6/22 (27.3)	10/41 (24.4)
CD34	211/274 (77)	29/61 (47.5)	100/135 (74.1)	111/139 (79.9)	7/14 (50)	22/47 (46.8)	108/151 (71.5)	103/123 (83.7) *	11/22 (50)	18/39 (46.2)
CD45	231/277 (83.4)	63/63 (100)	117/138 (84.8)	114/139 (82)	14/14 (100)	49/49 (100)	126/153 (82.4)	105/124 (84.7)	22/22 (100)	41/41 (100)
CD10	254/277 (91.7)	27/61 (44.3)	123/138 (89.1)	131/139 (94.2)	8/14 (57.1)	19/47 (40.4)	143/153 (93.5)	111/124 (89.5)	8/22 (36.4)	19/39 (48.7)
CD20	155/248 (62.5)	NR	78/124 (62.9)	77/124 (62.1)	NR	NR	84/134 (62.7)	71/114 (62.3)	NR	NR
CD38	148/166 (89.2)	NR	77/85 (90.6)	71/81 (87.7)	NR	NR	80/86 (93)	68/80 (85)	NR	NR
CD19	276/277 (99.6)	NR	137/138 (99.3)	139/139 (100)	NR	NR	153/153 (100)	123/124 (99.2)	NR	NR
CD66c	42/67 (62.7)	NR	24/34 (70.6)	18/33 (54.5)	NR	NR	19/37 (51.4)	23/30 (76.7) *	NR	NR
CD2	NR	52/63 (82.5)	NR	NR	12/14 (85.7)	40/49 (81.6)	NR	NR	21/22 (95.5)	31/41 (75.6) *
CD7	NR	63/63 (100)	NR	NR	14/14 (100)	49/49 (100)	NR	NR	22/22 (100)	41/41 (100)
CD117	NR	12/63 (19)	NR	NR	1/14 (7.1)	11/49 (22.4)	NR	NR	1/22 (4.5)	11/41 (26.8) *
CD3cyt	NR	63/63 (100)	NR	NR	14/14 (100)	49/49 (100)	NR	NR	22/22 (100)	41/41 (100)
CD3mem	NR	45/63 (71.4)	NR	NR	10/14 (71.4)	35/49 (71.4)	NR	NR	18/22 (81.8)	27/41 (65.9)
CD4	NR	33/62 (53.2)	NR	NR	9/14 (64.3)	24/48 (50)	NR	NR	11/22 (50)	22/40 (55)
CD8	NR	43/62 (69.4)	NR	NR	9/14 (64.3)	34/48 (70.8)	NR	NR	16/22 (72.7)	27/40 (67.5)
CD5	NR	56/63 (88.9)	NR	NR	13/14 (92.9)	43/49 (87.8)	NR	NR	22/22 (100)	34/41 (82.9) *
CD1a	NR	25/61 (41)	NR	NR	7/14 (50)	18/47 (38.3)	NR	NR	10/22 (45.5)	15/39 (38.5)

* p <0.05.

NR: not rated; mem: membrane; cyt: cytoplasmic; B-cell ALL: B-cell acute lymphoblastic leukemia. T-cell ALL T: T-cell acute lymphoblastic leukemia.

* p<0.05.

**Figure 4:**
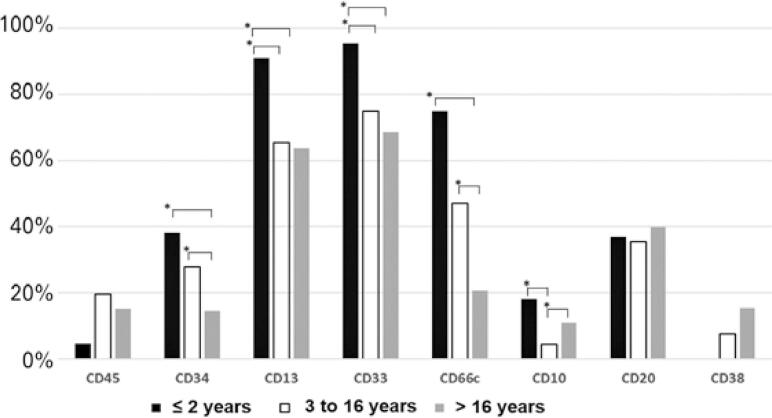
Percentage of B-cell ALL cases with negative expression of antigens by age group

Comparing the age groups in B-cell ALL, significant differences were observed in expression of CD10, CD13, CD33, CD66c and CD34 (Figure 4).

* p<0.05.

## DISCUSSION

The present study is the first research carried out in Bahia to characterize the immunophenotypic profile of acute leukemias, as of our knowledge. The data comprise most cases of leukemia diagnosed in the state of Bahia, since they were collected at a diagnostic reference center in the state.

Most cases were AML (57.3%), and less than what was reported by Salem et al.^(19)^(68.9%), who assessed a greater number of adult patients. Acute promyelocytic leukemia accounted to 17.6% of AML cases, a frequency higher than that reported by Ghosh et al.,^(20)^ who found 6%, and lower than that described by Salem et al^(19)^ (23%). According to the World Health Organization (WHO),^(5)^the frequency of APL in AML is 4-5%. In our study, morphological and immunophenotypic criteria were used to assess diagnosis of APL.

The median age (43 years) in the AML group was very different from that reported by WHO (63 years). The incidence of AML is known to increase with age, and this difference could be explained by the fact that developed countries have a higher concentration of elderly population.^(21,22)^ A Brazilian study conducted by Capra et al.^(23)^found a very similar median age of 42 years. The frequency of AML was higher in females, 53.2% of cases (p=0.236), in contrast to what is reported in the literature.^(24-26)^

As to expression of aberrant markers in AML, positive expression was more frequent for CD7 (37.4%), followed by CD56 (18.4%). The present study demonstrated a higher frequency of CD7 than other investigations, ranging from 8.8% to 33%.^(16,27)^ CD7 expression in myeloblasts has been associated with failure to achieve complete remission and decreased survival.^(28,29)^ It is believed that CD7 is expressed in early stages of hematopoietic ontogeny and has been associated with expression of precursor antigens.^(28,30)^ Corroborating this statement, we found the expression of markers linked to cellular immaturity, such as HLA-DR in 91.4%, CD117 in 97.8%, and CD34 in 87.6% of CD7 positive AML cases. Myeloperoxidase (MPO) appears in more mature stages and was demonstrated in only 40% of cases.

As regards to CD56, the literature reports a frequency between 11.6% and 27.6%.^(26,27,31)^ In the present study, 18.4% of cases expressed CD56. Raspadori et al.^(32)^ emphasized this aberrant expression is related to a reduced probability of achieving complete remission and lower survival rates. Therefore, it is important to regularly assess the presence of CD56 in myeloid blasts and its expression must be considered in the therapeutic strategy.^(28)^

Comparing the phenotypes of non-APL AML and APL, in addition to HLA-DR and CD34, we found significant differences in the expression of markers CD13, MPO, CD15, CD64, CD2 and CD7. Thus, the professional in charge of making immunophenotypic diagnosis must ensure greater importance of these markers when reporting APL. According to the WHO, the main markers differentiating APL from other AML are HLA-DR, CD34, the homogeneous and strong expression of CD33, negative or weakly expressed CD15, and the expression of CD64 that is common in APL. CD2 expression in APL has been associated with FLT3-ITD mutation and may contribute to targeting therapies, such as tyrosine kinase inhibitors. CD56 expression was associated with an unfavorable prognosis.^(5)^

In acute leukemias secondary to CML, we observed 46% of cases progressed to B-cell ALL. The WHO reports a percentage of 20-30%. Cases of T-cell ALL and Natural Killer (NK) cells have already been reported.^(33)^ Studies reporting the frequency of acute leukemia secondary to CML are scarce. A Mexican study reported 35.3% of cases progressing to B-cell ALL.^(34)^

Acute myeloid leukemia has a complex genetic basis involving several genetic alterations.^(35)^ This fact may explain the different phenotypic alterations found among adults and children. CD13 and MPO were more expressed in adults in non-APL AML, and CD117 was more expressed in adults in APL. The group of children aged ≤2 years had a high frequency of cases with negative expression for CD13 and CD33. This fact should be considered when choosing immunotherapy with anti-CD33 antibody, which is one of the most used in AML.^(36)^

Among the most common subtypes of leukemia, ALL stands out in the pediatric group.^(37)^ In our study, ALL account for 78.3% of acute leukemia in children. According to Siegel et al.,^(38)^ who carried out a study about the Hispanic and Latin population, ALL accounted for 78% of leukemia cases in children. In adults, the frequency of ALL was 27.6%. B-cell ALL accounted for (81.5%) of ALL cases, corroborating data reported by the WHO (80-85%). In T-cell ALL, 77.8% of cases were males, as described in most literature reports.^(39)^

Aberrant markers help to differentiate normal or reactive cells from leukemic cells and are important to evaluate minimal residual disease.^(17,40)^ Concerning the expression of aberrant markers in B-cell ALL, the present study found a positivity rate more frequent for CD66c (62.7%) followed by CD13 (33.3%) and CD33 (26.1%). CD66c is the most frequent aberrant marker in B-cell ALL;^(41,42)^ therefore, it is the most useful marker to study minimal residual disease. However, it is still not widely used and was added to B-cell ALL panels only a few years ago. For this reason, few studies have reported frequency of positive CD66c in B-cell ALL patients. Kalina et al.^(15)^ demonstrated CD66c expression in 43% of B-cell ALL cases, and Ismail et al.,^(43)^ in 51.8%. When analyzing CD13 and CD33 expression, the study presenting results more similar to ours was a Brazilian study carried out by Alves,^(44)^ with a frequency of 33% and 25%, respectively.

The phenotypic pattern of antigen expression reflects the gene expression of leukemic cells. When comparing the expression of aberrant markers in adults and children, we observed a significant difference for CD66c. This fact may be related to the expression of CD66c being associated with B-cell Philadelphia (Ph) + ALL, which is more frequent in adults.^(41)^ CD13 and CD33 were also more often expressed in adults.

CD34 is a transmembrane protein, widely used as a marker of hematopoietic stem cells. Its biological function is involved in inhibition of differentiation, expansion of hematopoietic stem cells, signaling transduction and adhesion.^(45)^ Studies showed that in pediatric ALL, the expression of CD34 is a good prognostic factor, while in adults it is related to a worse prognosis.^(46-48)^ CD34 expression was significantly higher in adults, and what may be associated with prognostic features of the genetic basis of the disease.

CD10 expression was significantly different between the pediatric and adult groups. The group of children had a higher frequency of negative CD10 in B-cell ALL. This difference can be explained by the fact that pro-B ALL is the most frequent leukemia in children aged <1 year, and it is less common in older children and adults.^(5)^ Pro-B ALL is characterized by negative CD10, and its most frequent molecular alteration is *KMT2A* t(4;11) rearrangement; this subtype has an unfavorable prognosis.^(49,50)^ The frequency of negative CD10 in B-cell ALL cases was 8.3%, close to 11.36%, as reported by Haddad et al.^(11)^

In T-cell ALL, the most frequent aberrant markers were CD10 (44.3%), CD13 (36.5%), CD33 (25.4%) and CD117 (19%). Sayed et al.,^(51)^ in Egypt, found a frequency of 45.9% for CD10, 4.4% for CD13, 10.1% for CD33 and 5.1% for CD117. In India, Garg et al.^(52)^ reported a frequency of 35.3% for CD10, 38.46% for CD33 and 42.28% for CD117. Comparing the phenotype of the pediatric and adult groups, significant differences were observed for expression of CD2, CD117 and CD5, possibly because the frequency of early T-cell precursor ALL is higher in adults.^(53)^

Studies showed the relation between an elevated leukocyte count with unfavorable prognosis, especially in B-cell ALL.^(54,55)^ When evaluating expression of antigens in different white blood count groups in T-cell ALL, a significant difference was observed in expression of CD2 and CD5. A study by Chopra et al.^(53)^ demonstrated the white blood count of early T-cell precursor ALL group is much lower than that of other T-cell ALL subtypes; what may explain the differences found in expression of CD2 and CD5 in white blood count groups in the present study.

## CONCLUSION

This work contributed to documenting the immunophenotypic profile of 796 new cases of acute leukemia in the state of Bahia. In contrast to the literature, the incidence of acute myeloid leukemia was slightly higher in women. Significant differences were found in several antigens evaluated when comparing the pediatric and adult groups. CD66c and CD7 were the most frequent aberrant markers in B-cell acute lymphoblastic leukemia and acute myeloid leukemia, respectively, and the frequency was higher as compared to the literature. To determine the positivity of these markers in a standardized fashion, it is necessary to evaluate the influence of the population and the criteria for standardization of analytical processes employed by laboratories. Based on these findings, future studies correlating the phenotypic profile with genetic characteristics and therapeutic response, including antigen-specific therapies, may be better targeted.
